# Interview with Prof. Claudio L.A. Bassetti, President of the European Academy of Neurology

**DOI:** 10.25122/jml-2022-1031

**Published:** 2022-10

**Authors:** Alexandra Gherman, Stefana-Andrada Dobran

**Affiliations:** 1 RoNeuro Institute for Neurological Research and Diagnostic, Cluj-Napoca, Romania; 2 Sociology Department, Babes-Bolyai University, Cluj-Napoca, Romania

Professor Dr **Claudio Bassetti** currently serves as Professor of Neurology, Director of the Department of Neurology of the Inselspital and Dean of the Medical Faculty at University of Bern, Switzerland, and President of the European Academy of Neurology.

This is an adapted interview upon the occasion of being conferred the Doctor Honoris Causa Award from Iuliu Hatieganu University of Medicine and Pharmacy, September 21^st^, 2022, Cluj-Napoca, Romania.

With me here is Professor Dr **Claudio Bassetti**, Professor of Neurology, Director of the Department of Neurology, Dean at the Medical Faculty University Hospital in Bern, Switzerland, and President of the European Academy of Neurology.

**Q:** Prof. Bassetti, welcome to Cluj-Napoca, and congratulations on the Doctor Honoris Causa [award] received from the Iuliu Hatieganu University of Medicine and Pharmacy for your long-lasting involvement and collaboration with the Neurosciences Department. And for the beginning, I would so much like to know what this award means for you.

**A:** It means a lot, it is great recognition; I am very honoured! I am also very pleased to be in Cluj-Napoca. As you mentioned, it is a longstanding clinical and scientific partnership, but I would like to strengthen, also a longstanding friendship with the Department, with Professor Dafin Muresanu, his team, and, I would say, the entire community in Romania. I had the possibility, over the years, to visit different places with him and through him, and so I feel very close to the entire neuroscience community in Romania.

**Q:** So, congratulations once again on this award!

**A:** Thank you!

**Q:** And talking about research, considering your unique background in sleep research and in disorders, sleep disorders, what do you think are the following most exciting developments in this field?

**A:** Well, you know, there is a big question about the function of sleep. It seems a little bit strange, to some extent, if you think that the reason why we sleep still remains somewhat unknown or, at least, there are still discussions and debates about this. So, I think we start understanding better the reasons that we sleep, and I should mention that, as a neurologist, it is important to stress the fact that we sleep mainly, not only, for the brain, so there is something about sleep that is essential for our brain. Memory and learning functions are enhanced by sleep, but also the removal of toxic proteins from the brain (a kind of "brain wash") are facilitated during sleep. We have also understood from several clinical studies that sleep loss is associated with a multitude of negative consequences for our body, brain and mental health. Personally, I have been involved for example in studies showing that sleep loss and sleep apnea, increase the risk of stroke and dementia. As a consequence, it will be important for the future to understand how we can promote sleep to foster our health and by this have better lives, better body, brain and mental functions.

**Q:** So, sleep definitely is a must.

**A:** Sleep is a must. Not alone, obviously. We cannot sleep ourselves completely into health. But sleep together with diet, physical activity, cognitive activity and social interactions,are important pillars for our overall health.

**Q:** Is there "too much sleep"?

**A:** There is sometimes "too much sleep" and, paradoxically, studies have suggested a link between the report of long sleep hours with and increased mortality. This may appear at first glance a paradox because I just said that sleep is good. The above mentioned observation is however explained by a different hypothesis, that of long sleep representing a symptom of an underlying disease.

**Q:** So, we should stick to the 8 hours average.

**A:** Well, average, it is true. I mean, there are recent studies actually confirming that this is what most people do, between 7 and 9 [hours]. There are few exceptions, so-called long and short sleepers. Unfortunately, in the last 30 years we observed an increasing tendency in the general population to sleep less and less. Noteworthy, the percentage of people taking regularly sleeping pills is conversely increasing. Society is developing sleep behaviors that are in contrast with what sleep expert actually suggest.

**Q:** How has COVID-19 changed the landscape of neurological care from your point of view?

**A:** Well, this is obviously a very complicated and very important question. First of all, we actually…

**Q:** Because COVID-19 was very related to neurological problems.

**A:** Sure, sure. I was going to say. Obviously, we all suffered from the fact that resources, human resources, and also, I would say, medical and hospital resources [...] diverged from neurological care to general care – this happened also in my country, Switzerland. Now, as you alluded to, we know that neurological complications are very frequent; depending on which statistics you look at, up to 20–30% of patients with COVID do have neurological complications. As President of the European Academy of Neurology (EAN), I also launched a systematic study, an international study, which we called ENERGY, on the neurological complications of COVID. So, we learned that there is a brain affinity of the virus, and then we also started understanding that long COVID is also a disease or a condition that is frequently accompanied by neurological problems, but also with a frequency association also with mental disorders. This association stresses once more the need for a good collaboration between neurologists and psychiatrists. It was indeed one of my strategic actions of my presidency to foster the collaboration between these two specialties.

**Q:** Should we, people … maybe who went through the disease, be more careful or go into certain… analyses, should we go to the doctor especially to check?

**A:** Well, no, I don't think so. It depends really on the presence or not of residual symptoms – If you have smell problems, if you have cognitive problems. There are some studies showing that long COVID may be accompanied by cognitive and other brain dysfunctions. Then, obviously, yes. Fatigue is a very frequent symptom, [as well as] sleepiness, sleep disorders, [...] and smell problems I mentioned already. So, if you have residual symptoms, you should check how to best deal with them, and the complexity of long COVID is that you have mental and brain disorders together.

**Q:** So, nothing hidden, there is some... some symptom to be felt if…

**A:** Yes, usually …usually you should have some perception of a disturbance, and then you should look for a specialist, yes.

**Q:** Please tell our audience more about the IGAP project, particularly the European Academy of Neurology's Brain Health Strategy you have spearheaded.

**A:** Well, yes, I would like to say that 2022 is really the year of neurology and neurological disorder. Why? Because for the first time, the WHO has decided to recognize neurological disorders as a public health priority. Why is this the case? Well, first, because we start having very good data, international scientific data showing that the frequency of neurological disorders is very high. We say that at least one person out of three, in the course of its life, of his life, her life, will develop a neurological disorder. So, it's very high. And this is really somewhat new in the landscape of scientific evidence. The second is that we have understood in the last 5, 10, and 15 years that much of neurological disorders can be prevented. There are some estimations and recent publications saying that up to 40% of the phenomenon of stroke, a typical neurological disorder, or dementia, another neurological disorder, 40–50%, can be prevented. So, we have a tool to do something to decrease the burden of neurological disorders. I mentioned the frequency, I should also say the costs of neurological disorders, which are tremendous. And for all these reasons actually, the… And I should also mention the fact that the workforce for neurological disorders is still insufficient in many countries, not only in Asia, South America but even in Europe, in some areas of Europe. So, there are multiple reasons why actually the World Health Organization has launched the IGAP – Intersectorial Global Action Plan on epilepsy and neurological disorders, which actually puts neurological disorders on the map of, you know, the health authorities. And the EAN actually has launched a brain health campaign just to support and accompany this initiative, and actually, we want to promote this knowledge, and we want to support different national campaigns and initiatives that have started in Norway and in Germany. And in recent time I have also launched (and coordinated) an initiative in Switzerland, and I would be very happy to support also the start of such an initiative in this country.

**Q:** When you mention diseases that can be prevented, what do you mean by that? Do you mean lifestyle?

**A:** Well, as I mentioned, you can prevent them with a healthy diet, and you can prevent them by promoting good sleep and enough sleep; there are some disorders or diseases that should be treated early. You know, for instance, people do neglect the fact that if you start having cognitive decline, or dementia, you should treat cataracts, you should treat hearing loss. So, there is a number of measures and preventable approaches that may actually diminish, you know, the risk of these disorders. Also, mental activity, and physical activity, so there are a number of approaches and measures that one should take into account to try to prevent some diseases that may be then difficult to be treated.

**Q:** With what age? Starting with what age?

**A:** Well, you know, the brain health strategy of the WHO and the same of the EAN actually starts even before birth. So, the way … the mother, the pregnancy, the education you give to your children, the diet. I mean, we do know that non-communicable diseases, obesity, and increased diabetes, are becoming epidemics in areas in Europe and worldwide. So, I think it's actually an approach that starts from early on and should not be started, let's say, when you become demented. That's often a little bit too late or, I should say, too late, definitely. You still can promote brain health even if you get sick. I mean, we want to show that you can improve your life and also your quality of life if you started having a neurological disease or if you had a stroke, or if you start having cognitive decline. But obviously, the more you start before, the better you can get the results.

**Q:** Coming back to Romania, why do you think Romania needs a National Strategy for Cerebrovascular Disease?

**A:** Well, stroke is a very frequent and very devastating condition, and I observed, over the last 15 years, big support for the creation of stroke units, the use of thrombolysis, which is a treatment that can change, you know, the lives of patients with acute stroke, but this is not sufficient. Scientific evidence has shown in the last, I would say, 10 years that also thrombectomy, so endovascular treatment, can be very effective, so, I actually think that promotion of not only thrombolysis or stroke unit, but also of complex stroke centres where, you know, quite cumbersome treatment such as thrombectomy where you have a need for endovascular therapies, endovascular and neurological, these should be promoted and I welcome, you know, these steps and measures that have been started in this direction.

**Q:** And what dimensions of brain health should be included in such an endeavour? At which levels of our health system?

**A:** Well, it's a complicated question because I think the Intersectorial Global Action Plan, as the name says, stresses the fact that neurologists should partner with other professionals, medical professionals, some ideas came about the radiologists I mentioned; I mentioned also about the general practitioners that should be aware of the preventive measure. But it's also about the interprofessional measures with nurses and other, you know, health specialists. I'm thinking about also bioengineers and data scientists. Big data are becoming the clue for many approaches. So, I think neurologists and the care of neurological disorders should now include and embrace many other specialists, and this is what Intersectorial is about. As I mentioned before, there are areas and countries where there is an insufficient neurological workforce, so we are forced to use other specialists to deliver neurological care. I think about Africa; there are countries in Africa that have 0 (zero) neurologists, so I think the future is to really work together, including also the information of the general population. So, there is also an awareness that needs to be increased about neurological disorders. A lot to be done, but I think this year, as I said, is a historical year, and I am happy to be in Romania also to promote, you know, the prevention of neurological disorders, brain health, and everything that turns around these conditions.

**Q:** We are also happy to have you here, happy and honoured, and thank you for taking the time to talk to us and to offer us your insights on this fantastic subject!

**A:** Thank you very much! It's a very interesting occasion for me to be here with all of you. Thank you!

**Q:** Thank you!

